# Genetic Heterogeneity of Undifferentiated Pleomorphic Sarcoma: Is There Potential for Targeted Therapy?

**DOI:** 10.3390/cancers17223613

**Published:** 2025-11-10

**Authors:** Ekaterina A. Lesovaya, Timur I. Fetisov, Beniamin Yu. Bokhyan, Maria A. Senchenko, Dmitry V. Rogozhin, Varvara P. Maksimova, Anna N. Demko, Gennady A. Belitsky, Marianna G. Yakubovskaya, Kirill I. Kirsanov

**Affiliations:** 1 Department of Chemical Carcinogenesis, N.N. Blokhin National Medical Research Center of Oncology, Ministry of Health of Russia, Moscow 115478, Russia; lesovenok@yandex.ru (E.A.L.); timkatryam@yandex.ru (T.I.F.); beniamin-bokhyan@mail.ru (B.Y.B.); senchenko.mariia@yandex.ru (M.A.S.); pathol.777@mail.ru (D.V.R.); lavarvar@gmail.com (V.P.M.); belitsga@mail.ru (G.A.B.); kkirsanov85@yandex.ru (K.I.K.); 2 Oncology Department, Ryazan State Medical University Named After Academician I.P. Pavlov, Ministry of Health of Russia, Ryazan 390026, Russia; naetochka@yandex.ru; 3 Institute of Medicine, RUDN University, Moscow 117198, Russia

**Keywords:** undifferentiated pleomorphic sarcoma, genetic heterogeneity, mutation, amplification, chromosomal translocation, signaling alteration, immunotherapy

## Abstract

Undifferentiated pleomorphic sarcoma (UPS) is the most morphologically and genetically heterogeneous form of soft tissue sarcoma. UPS tumors can exhibit a wide range of genetic abnormalities, including activating and inactivating mutations, gene amplifications, chromosomal translocations, and copy number variations. Owing to this extensive genetic heterogeneity, no UPS-specific therapeutic targets have yet been validated, complicating diagnosis, prognosis, and the selection of targeted treatment strategies. Currently, immune checkpoint inhibitors (targeting PD-1, PD-L1, and CTLA-4) are the only validated targeted therapy for UPS, reflecting the frequent mutational events that activate immune response pathways. Because molecular genetic profiling alone provides limited prognostic value for chemoresistance in UPS, the development of experimental ex vivo and in vitro testing approaches may help to identify and exclude potentially ineffective targeted therapies.

## Simple Summary

Despite advances in multitargeted anticancer drugs and personalized treatment approaches, the management of soft tissue sarcoma remains challenging. Among the most difficult subtypes to diagnose and treat with targeted therapies is UPS, owing to its high degree of genetic heterogeneity. This review summarizes recent findings on the genetics and epigenetics of this sarcoma subtype and discusses the potential application of various targeted therapies.

## 1. Introduction

Undifferentiated pleomorphic sarcoma (UPS), formerly known as malignant fibrous histiocytoma, is a heterogeneous group of pleomorphic sarcomas that lack a definable line of differentiation and specific molecular features and thus remain a diagnosis of exclusion [1]. This subtype of soft tissue sarcoma (STS) is an aggressive malignancy characterized by a high metastatic potential and limited responsiveness to current therapeutic modalities. Therefore, the identification of specific diagnostic and prognostic markers, along with the development of novel targeted therapeutic strategies, is urgently needed.

UPS typically occurs in late adulthood, most commonly between 50 and 70 years of age, with a higher prevalence among white males [1]. The most frequent sites of UPS development are the lower extremities—particularly the thighs—followed by the upper extremities and the retroperitoneum [2,3,4,5]. Clinically, UPS usually presents as a painless, slowly enlarging mass. Microscopically, it exhibits considerable morphological variability and is generally composed of highly pleomorphic cells. Spindle cell, round cell, and epithelioid variants have been described, and multiple histological components may coexist in the same tumor. The neoplastic cells often form fascicles or storiform structures; however, in some cases, no distinct growth pattern can be recognized. Additional histopathological features include high mitotic activity, areas of necrosis, and variable degrees of chronic inflammation [6].

Immunohistochemical (IHC) findings in UPS are generally limited and non-specific. The primary role of immunohistochemistry in UPS diagnosis is to exclude other, more specific sarcoma subtypes. Typically, UPS lacks expression of distinct lineage-specific markers but shows diffuse positivity for vimentin and variable or focal expression of CD68, smooth muscle actin (SMA), and CD34, all of which are considered low-specificity markers [7]. Over the past decades, numerous studies have identified various genetic and epigenetic changes associated with UPS; however, further comprehensive analyses are required to validate these molecular abnormalities as potential diagnostic markers and to refine criteria guiding therapeutic decision-making. In particular, several transcriptomic analyses of large STS cohorts have failed to clearly distinguish UPS from other STS subtypes, such as leiomyosarcoma (LMS). Therefore, a comprehensive analysis of recent molecular genetic studies, case reports, and findings from emerging optimized therapeutic approaches is essential for identifying molecular and genetic characteristics of UPS that may hold prognostic and therapeutic significance.

## 2. Molecular Genetic Heterogeneity of UPS

Owing to the absence of distinctive morphological or molecular features, UPS remains a diagnosis of exclusion in clinical practice. Specifically, myxofibrosarcoma (MFS) has been reclassified as a separate entity from UPS based on its fibroblastic differentiation and characteristic myxoid stroma; however, from a genetic standpoint, UPS and MFS are still considered part of the same sarcoma group. According to the current WHO Classification of Soft Tissue and Bone Tumors, UPS lacks specific gene transcripts and is characterized by a highly unstable genome [1,8].

Genetic aberrations in UPS—including chromosomal translocations, mutations, and deletions—are numerous but non-specific and may vary considerably among individual cases. Therefore, the significance of data derived from single case reports should not be overlooked. The karyotype of UPS cells is typically complex, with multiple chromosomes affected simultaneously. Reported chromosomal translocations include t(1;2), t(1;3), t(1;7), t(1;10), t(1;17), t(2;3), t(5;10), t(5;11), t(5;17), t(6;8), t(6;10), t(7;10), t(9;10), t(10;11), t(10;12), t(11;17), and t(15;21) [8,9,10]. Recently, isolated cases of UPS harboring *NTRK* rearrangements—including *TMTC–NTRK3*, *DCTN1–NTRK1*, and *SARM1–NTRK1*—have been reported in both adult and pediatric patients [11,12,13,14,15]. In a retrospective study, the TRIO::TERT fusion gene was identified in patients with UPS [16]. This finding was further supported by evidence from additional studies involving several non-translocation-related STS, such as MFS, pleomorphic rhabdomyosarcoma, and dedifferentiated liposarcoma, including UPS. Multiple *TRIO* fusions involving different partners and *TERT* exons were described in these studies, including *TRIO(ex33)–TERT(ex2)*, *TRIO(ex33)–TERT(ex3)*, *TRIO(ex34)–CDH18(ex2)–TERT(ex2)*, *TRIO(ex34)–CDH18(ex2)–TERT(ex3)*, *TRIO(ex33)*–*LINC01504(intron2)*, *TRIO(ex33)*–*LINC01504(intron3)*, and *TRIO(ex33)*–*LINC01504(exon4)* [17]. In another study, a similar fusion—lacking MDM2 amplification—was reported in a case of spindle cell liposarcoma [18]. Because TRIO fusions have been detected in tumors of various histological types, it has been proposed that these changes do not represent primary oncogenic drivers but instead contribute to tumor progression as secondary oncogenic events [17].

An *EML4*–*ALK* gene rearrangement associated with brain metastasis was reported in a case of primary malignant fibrous histiocytoma of the lung in a 59-year-old male patient [19]. ALK rearrangements, particularly the *EML4*–*ALK* fusion, are well documented in non-small cell lung cancer [20,21]. Therefore, this tumor may be related to a sarcomatoid carcinoma of the lung.

Integration of multiple omics datasets has advanced the genetic profiling of UPS. In a study analyzing 19 UPS tumors, including 2 paired recurrent and re-recurrent samples, 66 fusion genes were identified, of which 10 were novel. Specifically, retinoblastoma (RB1) fusions—such as *RB1-RNASEH2B*, *RB1-FGF14-AS1*, and *E2F6-FKBP4*—were observed in two tumor samples and correlated with increased Rb/E2F signaling activation [22]. Additional targeted fusions included pseudogene-related fusions (*CIC-DUX4L8* and *EIF2AK4-ANXA2P2*), as well as *PDGFRA-MACROD2* and *NCOR1-MAP2K1*. Other rare fusion genes reported in primary UPS included *CLTC-VMP1*, *FARP1-STK24*, and *PRDM10* fusions with *MED12* and *CITED2* partners [14,21,22]. Since the release of the 2020 WHO Classification, tumors harboring CIC rearrangements are recognized as a distinct entity—CIC-rearranged sarcoma [1]. However, the biological significance of most other identified fusion transcripts remains largely unknown.

Importantly, positive regulatory domain (PRDM) proteins play critical roles in cell proliferation, differentiation, and malignant transformation. PRDM10-containing fusion genes have been identified in low-grade UPS and are present in approximately 5% of cases, representing a clinically important UPS subset [23,24,25]. Additionally, in secondary radiation-induced UPS following primary breast cancer, a novel *COL3A1-GULP1* fusion (*COL3A1*: exon23–*GULP1*: exon5) was detected [26].

Both loss-of-function and gain-of-function mutations, as well as gene amplifications, are common genetic abnormalities in UPS; however, their clinical correlations remain under investigation. Several growth factors have been shown to be overexpressed in UPS and may serve as potential therapeutic targets. For example, a tumor subgroup exhibiting increased *IGF2* and *FGFR3* expression could warrant more aggressive treatment strategies. Despite these findings, no validated prognostic markers currently exist for UPS [27]. Amplifications and activating mutations of *PDGFRA*, *PDGFRB*, and *EGFR* have been reported in cardiac and rare intracranial UPS, providing a rationale for exploring therapies targeting PDGF receptors and EGFR [28,29]. Additionally, leucine-rich repeat-containing protein 15 (LRRC15), a target of TGF-β, is frequently overexpressed in UPS cells. LRRC15 mediates cell–cell and cell–matrix interactions and has emerged as a promising anticancer target owing to its high expression in mesenchymal-derived tumors, including UPS [30,31]. Increased expression of *WWTR1* and *YAP1* has also been observed in 50–60% of sarcomas, predominantly in UPS and dedifferentiated liposarcoma [32].

Several amplifications and activating mutations in genes commonly associated with other STS subtypes have been identified in UPS. Specifically, mutations and amplifications in *KIT*, *KRAS*, *PDGFRA*, *PDGFRB*, *PIK3CA*, *AKT*, *AXL, MMP13,* and *WNT7B* have been reported [10,29,33,34,35,36,37,38,39]. Amplification of *VGLL3*, which encodes a mechanosensitive transcriptional regulator, has also been observed in UPS [40]. Comprehensive genomic profiling has revealed six recurrent genomic alterations across UPS tumors of different anatomical sites: *TP53* R248W, *ATR* I2435V, *GNAS* P423H, *MKI67* A1493T, *PDCD11* Q838H, and *SF3B1* A263V. In one case of orbital UPS, additional alterations in *NOTCH1*, *PCLO*, *MYST1*, and *NPM1* were detected [41].

Bioinformatic analysis of TCGA data and the gene expression profile GSE21050, combined with immunohistochemical evaluation of a tissue microarray (TMA) and in vitro validation, revealed that high expression of adenosine monophosphate deaminase 2 (*AMPD2*)—a key enzyme in purine metabolism—is associated with poorer patient outcomes across independent cohorts, potentially by promoting UPS cell proliferation [42]. In another study, overexpression of serine/threonine-protein kinase 13 (Plk1) and the DNA replication inhibitor geminin was identified as biomarkers of poor prognosis and provided novel insights into UPS biology [43]. Additionally, in 24 UPS samples, strong negative correlations were observed between the multidrug resistance genes *ABCB1* and *ABCG2*, while a positive correlation between MVP expression and favorable response to doxorubicin/gemcitabine therapy was reported [44].

In addition, UPS frequently harbors inactivating mutations and deletions in tumor suppressor genes, including *CDKN2A* (mutations and deletions), *TP53*, *CSF2RB*, *RB1*, *PTEN*, *ATM,* and *ATRX*, but lacks a single defining driver mutation [10,39,45,46,47,48]. Notably, loss of function in *RB1* and *TP53* leads to activation of SKP2 and increased cell proliferation, suggesting that SKP2 inhibition may represent a potential therapeutic strategy [49].

In a study of 60 UPS cases using cDNA microarray analysis, a 300-gene signature (11% FDR) was identified. The most upregulated gene clusters comprised cathepsins and regulators of protein degradation, inflammation, cell motility, and proliferation [50]. Interestingly, increased expression of the cytoskeletal component COL6A3 and biglycan (BGN) was associated with a favorable prognosis in studies of 46 and 38 UPS patients, respectively, despite the potential oncogenic role of the *COL6A3-GULP1* fusion [26,51]. Furthermore, dysregulation of alternative splicing events has been implicated in UPS pathogenesis and progression. Specifically, exon skipping in the *EWSR1* gene correlates with poor prognosis, although this finding requires further validation [52].

Copy number variations (CNVs) in UPS have been reported as follows: gains at 1p36.33–p31.3, 1q21.2–q24.3, 4p16.3, 5p15.33–p13.1, 7p22.3, 7p15.2–7p11.2, 7q32.1–q32.2, 9q34.3, 14q11.2, 14q32.33, 16p13.3, 17q12, 17q21.33, 17q23.3, 19p13.3, 19q13.11–q13.2, 19q13.42, 20q11.21–q13.33, 21q22.3, and X; losses at 1q32.1, 2p25.3, 2q36.1–q37.3, 8p23.3, 9p24.2–9p22.3, 9p21.3–p21.1, 10q21.1–q23.2, 11q22.3, 13q12.11–q31.1, 13q33.3, 16q11.2, and 16q23.1; and amplifications at 1p36, 1p32, 1q21–q23, 1q32, 3q26, 4q, 5p, 6q23, 7q, 8p23.1, 8q21.2–q22, 8q24, 9q31–q34, 10q26, 11q, 12p, 12q13–q15, 17q12, and 20q. Among these, deletion of 13q, particularly 13q14–21, was the most common and has recently been identified as the most frequent copy-number alteration in UPS [8,53].

Near-haploidization, characterized by the loss of one copy of chromosomes, is relatively rare in most tumors but may represent a specific feature of UPS. However, clinical validation of this phenomenon remains limited because only a single study has been reported in the literature. Whole-genome and transcriptome sequencing of two UPS samples revealed chromosomal rearrangements in the form of copy number variants, specifically affecting *SMC1A*, a gene encoding a component of the cohesin complex and a key regulator of the S-phase [54].

## 3. Epigenetic Alterations in UPS

UPS is not defined by specific epigenetic changes; however, subsets of epigenetic regulators and microRNAs (miRNAs) have been reported. Genome-wide studies indicate that UPS exhibits aberrant DNA methylation patterns. In particular, DNA methyltransferase DNMT3B is overexpressed in UPS and is associated with poor prognosis. Patient samples also show substantially elevated methylation of histones H3K4me3 and H3K9me3 compared with normal muscle tissue. However, current DNMT inhibitors, including 5-aza-2ʹ-deoxycytidine and nanaomycin A, are ineffective in UPS owing to unfavorable safety profiles [55,56].

MiRNA profiling has been investigated in several UPS studies. In one study of 10 high-grade UPS samples, miRNA microarray analysis identified differentially expressed miRNAs, along with their target genes, including miR-199b-5p, miR-320a, miR-199a-3p, miR-126, and miR-22, targeting *IMP3*, *ROR2*, *MDM2*, *CDK4*, and *UPA*. In a subsequent series of 27 UPS samples, these findings were validated using quantitative polymerase chain reaction (PCR) [57]. Another study identified a distinct subset of differentially expressed miRNAs, predominantly with tumor suppressor functions, including miR-451, miR-1260, miR-1274a, miR-34a, miR-152, miR-199b-5p, and miR-320a [58]. Interestingly, in UPS samples with mutated BRAF V600E and KRAS G12D and increased MAPK signaling, higher levels of mature miRNAs were detected. In vitro and in vivo experiments showed that mutations in Dicer, a key enzyme in miRNA biogenesis, cooperated with oncogenic KRAS and BRAF mutations to promote tumor progression in vivo [59].

In addition, long noncoding RNAs (lncRNAs) act as tissue-specific regulators of gene expression, and their upregulation can promote carcinogenesis, particularly sarcomagenesis. Data on the role of lncRNAs in UPS are limited; however, an in vivo study demonstrated that upregulation of Nuclear Enriched Abundant Transcript 1 (NEAT1) may facilitate UPS metastasis to the lungs [60].

To date, no epigenetic therapy has been proven effective, nor have any of the described epigenetic markers demonstrated prognostic value in UPS. Because epigenetic regulation directly influences chromatin organization, it can contribute to genomic instability and tumor progression, or conversely, enhance sensitivity to standard therapies as a result of drug-induced epigenetic modifications. Further studies in this field are therefore warranted.

## 4. Changes in UPS Signaling and Associated Therapeutic Approaches

**Figure 1:**
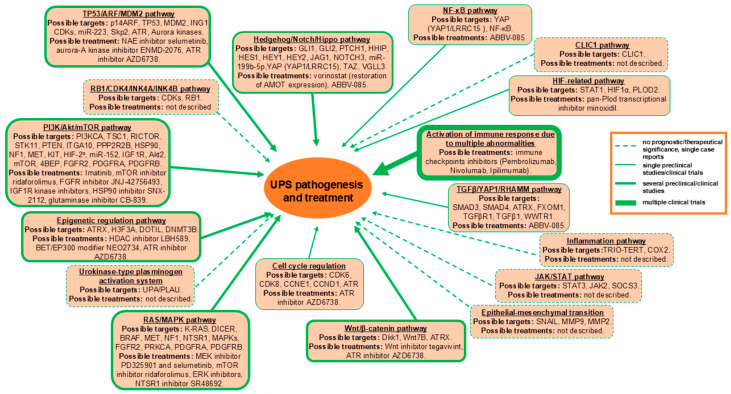
Potential prognostic/therapeutic targets in UPS.

UPS remains poorly understood both clinically and molecularly, largely owing to its intrinsic phenotypic and cytogenetic complexity. As described above, UPS may harbor multiple genetic and epigenetic abnormalities, yet no specific prognostic or predictive biomarkers have been definitively established. The complex genetics of UPS limit the effectiveness of standard chemotherapy, leaving surgical resection and adjuvant radiotherapy as the primary treatment options. Therefore, identifying more effective therapies for UPS patients is urgently needed. Although numerous studies have investigated aberrant signaling in UPS cells, only a few of the most common signaling alterations have been characterized. The therapeutic targets with potential clinical validation and the corresponding possible therapies are summarized below and shown in Figure 1.

Aberrations in the RB and TP53 genes result in decreased activation of their corresponding signaling pathways. For example, in most tumors without TP53 mutations, deletion or silencing of the *p14ARF* gene, a negative regulator of MDM2, has been observed, highlighting the potential role of the p14ARF–MDM2–TP53 axis in UPS pathogenesis. Restoring TP53 activity may represent a potential therapeutic approach for UPS. Alterations in RB/TP53 have been shown to shift cancer cell survival toward the oncogenic protein SKP2. TMA data revealed a correlation between loss of RB and TP53 expression and positive SKP2 expression. Inhibition of SKP2 using the neddylation-activating enzyme (NAE) inhibitor pevonedistat suppressed proliferative activity in both patient-derived UPS cells and murine models, providing a rationale for novel systemic therapies [59]. TP53 has been shown to negatively regulate aurora kinases, and loss of p53 results in increased aurora kinase levels, which could serve as a target for therapy. In particular, ENMD-2076, an aurora-A kinase inhibitor with anti-angiogenic properties, demonstrated activity in a phase II study in patients with STS, including three patients with UPS [61,62].

The RAS/mitogen-activated protein kinase (MAPK) and phosphoinositide 3-kinase (PI3K)/mammalian target of rapamycin (mTOR) pathways are frequently activated in UPS, contributing to tumor progression and poor prognosis [63]. Components of these signaling pathways represent potential therapeutic targets. In preclinical studies, the MEK inhibitor PD325901 slowed tumor growth in vivo [64]. Several clinical trials have evaluated RAS/MAPK inhibitors in large cohorts of patients with STS; however, no responses to sorafenib were observed in UPS [65]. The MEK inhibitor selumetinib induced a partial response in 1 of 2 UPS patients [66]. Similarly, in a phase II study of the mTOR inhibitor temsirolimus, none of the eight UPS patients achieved a response [67]. Notably, the mTOR inhibitor ridaforolimus elicited a partial response in one UPS patient [68]. It is important to note that in vitro, HRAS G12V-driven UPS cells rapidly developed resistance to treatment with either a single MEK or ERK inhibitor; however, combination therapy effectively overcame this resistance [69].

Immunohistochemical analyses of UPS samples frequently reveal phosphorylated AKT (p-AKT), p-mTOR, p-S6RP, p-4EBP, as well as HGF, c-Met, and MEK/ERK, supporting the involvement of PI3K/AKT/mTOR and c-Met signaling in UPS pathogenesis. In preclinical studies, HSP90 overexpression was associated with elevated levels of p-AKT, p-mTOR, and p-S6RP, suggesting its potential as both a poor prognostic marker and a therapeutic target [70]. Notably, the selective HSP90 inhibitor SNX-2112 demonstrated antitumor activity in vitro in UPS cells by inducing apoptosis and autophagy, inhibiting mTOR phosphorylation, and suppressing MAPK signaling [71,72].

Another signaling pathway with oncogenic potential in UPS is the Janus kinase (JAK)–signal transducer and activator of transcription (STAT) pathway, which regulates gene transcription and cell proliferation. In a study of 79 UPS patient samples, phosphorylated STAT3 and its negative regulator SOCS3 were detected in 59.7% and 55.8% of samples, respectively, correlating with favorable and unfavorable prognosis. These findings suggest that STAT3 may serve both as a prognostic marker and a therapeutic target in UPS [73].

Additionally, loss of ATRX expression occurs in 20–30% of UPS cases and confers selective sensitivity to Wnt pathway activation. The Wnt signaling inhibitor tegavivint reduces UPS cell viability, representing a potential therapeutic approach for ATRX-deficient UPS [48].

Aberrant activation of Hedgehog and Notch signaling also contributes to UPS proliferation, involving multiple effectors, including *NOTCH3*, *JAG1*, *GLI1*, *PTCH1*, *HHIP*, *HES1*, *HEY1*, and *HEY2*. Deregulation of the Hedgehog-linked tumor suppressor Hippo pathway may further promote tumor progression [74,75,76]. Notably, YAP1, a transcriptional regulator and central effector of the Hippo pathway, is aberrantly stabilized in UPS owing to epigenetic silencing of its inhibitor Angiomotin (AMOT) and Hippo kinase copy number loss. In vivo experiments demonstrated that YAP1–TGFβ crosstalk enhanced cell proliferation and motility [40,74,75,77]. Treatment with epigenetic modulators vorinostat and JQ1 restored AMOT expression and Hippo pathway signaling, highlighting a potential therapeutic strategy warranting further investigation [78]. The TGF-β–linked membrane protein LRRC15 is expressed in several STS, including UPS, highlighting the urgent need for effective therapies. The antibody–drug conjugate ABBV-085, developed to target LRRC15, demonstrated an overall response rate of 20% in a clinical trial of ABBV-085 monotherapy in patients with osteosarcoma or UPS [31,79]. In addition, YAP1 contributes to the activation of NF-κB and VEGF signaling, and its inhibition is related to the modulation of the unfolded protein response (UPR), suggesting these pathways as therapeutically relevant targets [77,80,81]. Overexpression of proteins in NF-κB, VEGF, and UPR pathways may also serve as biomarkers of therapeutic sensitivity. Notably, higher levels of CREB3L1 correlate with increased doxorubicin sensitivity in in vivo studies [82].

A distinct therapeutic approach for UPS is immunotherapy owing to its status as a highly mutated STS subtype, which elicits a pronounced immune response. UPS tumors often exhibit elevated PD-L1 and PD-1 expression, increased T-cell infiltration (positive for CD3, CD8, CD127/IL7 receptor, CD99, CD68, CD10, and negative for TIGIT), and abundant tumor-associated macrophages (positive for CD163, ionized calcium-binding adaptor molecule 1 (Iba1), MSR1, CD204, and SIRPα), alongside a high tumor mutational burden (TMB-H) compared with other STS subtypes [38,83,84,85,86,87,88,89,90,91,92]. Anti-PD-1/PD-L1 drugs have been introduced into UPS therapy [89,93,94], and several clinical trials are ongoing, including: Pembrolizumab (PD-1) + Gemcitabine (pyrimidine antimetabolite) [87,95,96]; Toripalimab (PD-1) + Anlotinib (tyrosine kinase inhibitor, TKI) [90,97]; Carilizumab (PD-1) + Apatinib (TKI) [98]; Envafolimab (PD-L1) + Ipilimumab (CTLA-4) [99]; Nivolumab (PD-1) + Ipilimumab (CTLA-4) [100]; Pembrolizumab (PD-1) + Cyclophosphamide (alkylating agent) [101]; Pembrolizumab (PD-1) monotherapy [101]; checkpoint blockade inhibitors combined with radiation therapy [100,102,103,104]; Nivolumab (PD-L1) + Bempegaldesleukin (CD122-preferential IL-2 pathway agonist) [105,106]; Nivolumab (PD-L1) + Nab-sirolimus (mTOR inhibitor) [107,108]; Pembrolizumab (PD-1) + Eribulin (non-taxane microtubule inhibitor) [109,110]; and Pembrolizumab (PD-1) + Doxorubicin (topoisomerase II inhibitor) [111].

The SARC028 clinical trial demonstrated the efficacy of anti-PD-1 therapy in UPS [89,94]. This study also revealed a functional association between PD-L1 and CKLF-like MARVEL transmembrane domain-containing 6 (CMTM6), identifying CMTM6 as a novel regulator of PD-L1 expression and a prognostic marker associated with poor outcomes. Therapeutic strategies could potentially be guided by CMTM6 expression [112]. Additionally, the collagen-modifying enzyme procollagen-lysine,2-oxoglutarate 5-dioxygenase 2 (PLOD2), which is overexpressed in many tumors relative to normal tissues, has been shown to promote immune evasion in UPS, contributing to tumor metastasis and CD8^+^ T cell dysfunction. An in vivo study demonstrated that inhibition of PLOD2 reduced tumor growth and enhanced the efficacy of anti-PD-1 therapy, highlighting PLOD2 as a potential novel therapeutic target in UPS immunotherapy [113]. Several additional potential targets are currently being investigated in preclinical studies. The glutaminase inhibitor CB-839 showed therapeutic efficacy in a murine UPS model [114]. The epigenetic modifier NEO2734, which targets BET/EP300, demonstrated antitumor activity both in vitro and in vivo in murine UPS models characterized by activation of MYC-target pathways [115]. Furthermore, cotreatment of UPS xenografts in immunodeficient mice with a dual PI3K/mTOR inhibitor and an anti-IGF1R kinase inhibitor reduced tumor growth in vivo, while also decreasing UPS cell migration and invasion in vitro [116]. Using a comparative oncology approach, DNMT3B was identified as a potential therapeutic target; however, currently available anti-methylation drugs have not yet demonstrated effective clinical activity in UPS [56,117]. Another epigenetic modifier, the HDAC inhibitor LBH589, exhibited anticancer effects both in vitro and in vivo through downregulation of FOS-like antigen 1 (*FOSL1*) [118]. Additional potential targets include neurotensin receptor 1 (NTSR1) and human tumor endothelin 1 (TEM1), with the NTSR1 inhibitor SR48692 showing antitumor activity in vitro [119,120,121]. The ATM inhibitor AZD0156, in combination with the ATR inhibitor AZD6738, abolished UPS growth in vitro and in vivo [122]. Moreover, the FGFR inhibitor JNJ-42756493 demonstrated anticancer activity in cultured cells and patient-derived xenograft models of UPS, encompassing tumors with diverse phenotypes, prognoses, and molecular features [123].

UPSs are not commonly characterized by the expression of cancer-testis antigens (CTAs), which could potentially be targeted by T-cell receptor (TCR) gene therapy. Notably, a case was reported in which a patient with UPS received NY-ESO-1 TCR-transgenic T cells combined with dendritic cell vaccination and anti-PD-1 therapy, resulting in a durable antitumor response [124]. Similarly, MAGE-A3 TCR-engineered T cells, recognizing epitopes in MAGE-A3, could represent another potential immunotherapeutic approach for UPS [125].

Additional clinical trials investigating UPS therapy are summarized in Table 1.

## 5. Conclusions

UPS represents an STS subtype with highly heterogeneous genetics, creating considerable challenges in developing individualized treatment strategies. Numerous genetic and epigenetic aberrations in UPS have been investigated, but only a few may contribute to the development of targeted therapies for UPS. The accumulation of multiple genetic abnormalities may also result in hyperactivation of the immune system, making UPS a potential candidate for immunotherapy with checkpoint inhibitors, including anti-PD-1, anti-PD-L1, and anti-CTLA-4. Excluding ineffective therapies could improve treatment selection and patient outcomes. Developing experimental approaches for ex vivo and in vitro testing may help identify and eliminate therapies unlikely to benefit individual UPS patients.

## Figures and Tables

**Table 1:** Clinical trials on UPS treatment.

Type of Sarcoma	Therapy	Trial Registration Number
Unresectable or metastatic UPS	Anlotinib Hydrochloride + Toripalimab	NCT03946943
UPS	Envafolimab/Envafolimab + Ipilimumab	NCT04480502
UPS	Mecbotamab vedotin/Mecbotamab vedotin + Nivolumab	NCT03425279
Liposarcoma (LPS), leiomyosarcoma, UPS	Eribulin + Pembrolizumab	NCT03899805
Leiomyosarcoma, UPS	Pembrolizumab + Gemcitabine	NCT03123276
Recurrent or resectable UPS, dedifferentiated liposarcoma (DDLPS)	Nivolumab/Nivolumab + Ipilimumab and radiation therapy	NCT03307616
Advanced angiosarcoma, UPS	Propranolol + Pembrolizumab	NCT05961761
UPS, alveolar soft part sarcoma (ASPS)	Pembrolizumab + Melphalan + Dactinomycin	NCT04332874
Advanced UPS	Recombinant anti-PD-1 humanized monoclonal antibody (609A)	NCT05193214
Unresectable UPS and ASPS	Carilizumab + Apatinib	NCT04447274
Leiomyosarcoma, UPS, DDLPS	CSF1R Inhibitor (DCC-3014) + Avelumab	NCT04242238
Ewing sarcoma, osteosarcoma, UPS	Pembrolizumab + Cabozantinib	NCT05182164
Metastatic or unresectable UPS	doxorubicin and pembrolizumab	NCT06422806
Advanced STS	MAGE-12 Peptide Vaccine	NCT00020267
Advanced or metastatic STS	Brostallicin (PNU-166196A)/Doxorubicin	NCT00410462
Leiomyosarcoma, synovial sarcoma (SS), osteosarcoma, malignant peripheral nerve sheath tumor (MPNST), neurofibrosarcoma, desmoplastic small round cell tumor fibrosarcoma, ASPS, UPS, hemangiopericytoma, chondrosarcoma, epithelioid sarcoma, malignant mesenchymoma	Doxorubicin hydrochloride + Trabectedin	NCT01189253
Leiomyosarcoma, LPS, SS, MPNST, neurofibrosarcoma, ASPS, UPS, hemangiopericytoma, epithelioid sarcoma malignant mesenchymoma	Caelyx (pegylated liposomal doxorubin hydrochloride) + Ifosfamide	NCT00030784
Leiomyosarcoma, LPS, SS, fibrosarcoma, UPS, hemangiopericytoma	Soblidotin	NCT00064220
Leiomyosarcoma, LPS, SS, osteosarcoma, Ewing sarcoma, MPNST, neurofibrosarcoma, UPS, chondrosarcoma	Torisel + liposomal Doxorubicin	NCT00949325
UPS	XmAb^®^23104	NCT03752398
Multiple STS subtypes including adult UPS	Gemcitabine + Pazopanib	NCT01532687
Non small cell lung cancer, head and neck squamous cell carcinoma, pancreatic adenocarcinoma, colorectal cancer, UPS, solitary fibrous tumors, DDLPS	OKN4395/OKN4395 + Pembrolizumab	NCT06789172
High grade sarcoma, metastatic leiomyosarcoma, metastatic MPNST, metastatic SS, metastatic UPS, unresectable sarcoma, recurrent leiomyosarcoma, recurrent MPNST, recurrent SS, recurrent UPS	Sapanisertib (MLN0128 [TAK-228])	NCT02601209
Metastatic UPS and other multiple STS subtypes	Nivolumab/Nivolumab + Ipilimumab	NCT02500797
Recurrent adult STS, recurrent leiomyosarcoma, recurrent LPS, recurrent MPNST, recurrent UPS	MLN8237 (alisertib)	NCT01653028
Osteosarcoma, Ewing sarcoma, MFH, synovial fibrosarcoma, leiomyosarcoma	Reolysis (oncolytic virus)	NCT00503295
Advanced solid tumors, UPS, squamous cell carcinoma of the head and neck, carcinoma of the breast	ABBV-085	NCT02565758
Multiple STS subtypes including adult UPS	Epirubicin + Ifosfamide + Nivolumab	NCT03277924
Multiple STS subtypes including adult UPS	BO-112/BO-112 + Nivolumab	NCT04420975
Multiple STS subtypes including adult UPS	Talimogene laherparepvec (T-VEC) + Radiotherapy	NCT02923778
Fibrosarcoma, leiomyosarcoma, LPS, myosarcoma, histiocytic sarcoma, SS, lymphangiosarcoma, MPNST, UPS, DDLPS, pleomorphic rhabdomyosarcoma, malignant triton tumor	Radiotherapy + Sequential Doxorubicin and Ifosfamide	NCT03651375
UPS, SS, myxoid LPS and DDLPS	Sintilimab + Doxorubicin + Ifosfamide	NCT04356872
Extraskeletal myxoid chondrosarcoma, leiomyosarcoma, LPS, UPS	Ipilimumab + Nivolumab/Cabozantinib + Nivolumab + Ipilimumab	NCT05836571
Metastatic DDLPS, metastatic leiomyosarcoma, metastatic SS, metastatic UPS, DDLPS, unresectable leiomyosarcoma, unresectable SS, unresectable UPS	Peposertib + liposomal Doxorubicin	NCT05711615
Leiomyosarcoma, MPNST, UPS	Gemcitabine + Docetaxel + Pazopanib	NCT01418001
Leiomyosarcoma, LPS, SS, angiosarcoma, UPS, epithelioid sarcoma, MPNST, fibrosarcoma, pleomorphic rhabdomyosarcoma, endometrial stromal sarcoma, desmoplastic small round cell tumor	Doxorubicin + Ifosfamide	NCT06277154
Metastatic leiomyosarcoma, metastatic SS, metastatic UPS, advanced myxoid LPS, advanced STS, metastatic myxoid LPS, metastatic round cell LPS, metastatic STS, refractory leiomyosarcoma, refractory myxoid LPS, refractory round cell LPS, refractory STS, refractory SS, refractory UPS advanced leiomyosarcoma, advanced SS, advanced UPS, metastatic chondrosarcoma	Itacitinib	NCT03670069
LPS, leiomyosarcoma, UPS	Sunitinib	NCT00400569
UPS, leiomyosarcoma, LPS, SS, angiosarcoma	Olaratumab (Lartruvo) + Doxorubicin	NCT02451943 NCT02584309
Multiple STS subtypes including adult UPS	Ribociclib + Doxorubicin	NCT03009201

## Data Availability

The data for this study were collected from online resources only.

## Abbreviations

The following abbreviations are used in this manuscript:

ABCB1	ATP Binding Cassette Subfamily B Member 1
ABCG2	ATP Binding Cassette Subfamily G Member 2 (JR Blood Group)
AKT	AKT Serine/Threonine Kinase
ALK	Anaplastic Lymphoma Kinase
AMOT	Angiomotin
AMPD2	Adenosine Monophosphate Deaminase 2
ANXA2P2	Annexin A2 Pseudogene 2
ASPS	Alveolar Soft Part Sarcoma
ATM	Ataxia Telangiectasia Mutated
ATR	ATR Serine/Threonine Kinase
ATRX	ATRX Chromatin Remodeler
AXL	AXL Receptor Tyrosine Kinase
BCOR	BCL6 Corepressor
BET/EP300	BET and CBP/EP300 bromodomain
BGN	Biglycan
BRAF	B-Raf Proto-Oncogene, Serine/Threonine Kinase
CCNB	Cyclin B
CD	Cluster Of Differentiation
CDH	Cadherin
CDK4	Cyclin Dependent Kinase 4
CDKN2A	Cyclin Dependent Kinase Inhibitor 2A
CIC	Capicua Transcriptional Repressor
CITED2	Cbp/P300 Interacting Transactivator with Glu/Asp Rich Carboxy-Terminal Domain 2
CKLF	Chemokine Like Factor
CLTC	Clathrin Heavy Chain
CMTM6	CKLF Like MARVEL Transmembrane Domain Containing 6
COL3A1	Collagen Type III Alpha 1 Chain
CSF2RB	Colony Stimulating Factor 2 Receptor Subunit Beta
CTLA4	Cytotoxic T-Lymphocyte Antigen-4
DC	Dendritic Cells
DCTN1	Dynactin-1
DDLPS	Dedifferentiated Liposarcoma
DNMT3B	DNA Methyltransferase 3 Beta
DUX4L8	Double Homeobox 4 Like 8
E2F6	E2F Transcription Factor 6
4EBP	Eukaryotic Translation Initiation Factor 4E Binding Protein 1
EGFR	Epidermal Growth Factor Receptor
EIF2AK4	Eukaryotic Translation Initiation Factor 2 Alpha Kinase 4
EML4	Echinoderm Microtubule-Associated Protein-Like 4
ETV6	ETS Variant Transcription Factor 6
EWSR1	Ewing Sarcoma RNA Binding Protein 1
FARP1	FERM, ARH/Rhogef and Pleckstrin Domain Protein 1
FDR	False Discovery Rate
FGF	Fibroblast Growth Factor
FGFR	Fibroblast Growth Factor Receptor
FKBP4	FK506-Binding Protein 4
FOSL1	FOS Like 1, AP-1 Transcription Factor Subunit
GLI1	GLI Family Zinc Finger 1
GNAS	Guanine Nucleotide Binding Protein (G Protein), Alpha Stimulating Activity Polypeptide
GULP1	GULP PTB Domain Containing Engulfment Adaptor 1
HES1	Hes Family BHLH Transcription Factor 1
HEY	Hes Related Family BHLH Transcription Factor with YRPW Motif
HGF	Hepatocyte Growth Factor
HHIP	Hedgehog Interacting Protein
HSP90	Heat Shock Protein 90
Iba1	Ionized Calcium-Binding Adapter Molecule 1
IGF1R	Insulin Like Growth Factor 1 Receptor
IL-7	Interleukin-7
IMP3	IMP U3 Small Nucleolar Ribonucleoprotein 3
JAG1	Jagged Canonical Notch Ligand 1
JAK	Janus Kinase
KIT	KIT Proto-Oncogene, Receptor Tyrosine Kinase
KRAS	KRAS Proto-Oncogene, Gtpase
LINC	Linkers Of Nucleoskeleton and Cytoskeleton
LMNA	Lamin A/C
lncRNA	Long Non-Coding RNA
LPS	Liposarcoma
LRRC15	Leucine Rich Repeat Containing 15
MACROD2	Mono-ADP Ribosylhydrolase 2
MAGE-A3	Melanoma-Associated Antigen 3, Family Member A3
MAPK	Mitogen-Activated Protein Kinase
MDM2	Mouse Double Minute 2
MED12	Mediator Complex Subunit
MEK (MAP2K1)	Mitogen-Activated Protein Kinase Kinase 1
MFH	Malignant Fibrous Histiocytoma
MFS	Myxofibrosarcoma
MKI67	Marker of Proliferation Ki-67
MMP	Matrix Metallopeptidase
MPNST	Malignant Peripheral Nerve Sheath Tumor
MRI	Magnetic Resonance Imaging
MSR1	Macrophage Scavenger Receptor 1
mTOR	Mechanistic Target of Rapamycin Kinase
MVP	Major Vault Protein
MYST1	MYST Histone Acetyltransferase 1
NAE	NEDD8 Activating Enzyme
NCOR1	Nuclear Receptor Corepressor 1
NEAT1	Nuclear Enriched Abundant Transcript 1
NF-kB	Nuclear Factor Kappa B
NOTCH	Notch Receptor
NPM1	Nucleophosmin 1
NTRK	Neurotrophic Tropomyosin Receptor Kinase
NTSR1	Neurotensin Receptor 1
NY-ESO- 1	New York esophageal squamous cell carcinoma 1
PCLO	Piccolo Presynaptic Cytomatrix Protein
PCR	Polymerase Chain Reaction
PD1	Programmed Cell Death Protein 1
PDCD11	Programmed Cell Death 11
PDGFR	Platelet Derived Growth Factor Receptor
PD-L1	Programmed Cell Death Protein Ligand 1
PI3K	Phosphatidylinositol-4,5-Bisphosphate 3-Kinase
PIK3CA	Phosphatidylinositol-4,5-Bisphosphate 3-Kinase Catalytic Subunit Alpha
PLK1	Polo Like Kinase 1
PLOD2	Procollagen-Lysine,2-Oxoglutarate 5-Dioxygenase 2
PRDM	PR/SET Domain
PTCH1	Patched 1
PTEN	Phosphatase and Tensin Homolog
RB1	Retinoblastoma
RNASEH2B	Ribonuclease H2, Subunit B
ROR2	Receptor Tyrosine Kinase Like Orphan Receptor 2
S6RP	Ribosomal Protein S6
SARM	Sterile Alpha and TIR Motif Containing
SF3B1	Splicing Factor 3b Subunit 1
SIRPα	Signal Regulatory Protein Alpha
SKP2	S-Phase Kinase Associated Protein 2
SMC1A	Structural Maintenance of Chromosomes 1A
SOCS3	Suppressor of Cytokine Signaling 3
SS	Synovial Sarcoma
STAT	Signal Transducer and Activator of Transcription
STK24	Serine/Threonine Kinase 24
STS	Soft Tissue Sarcoma
TCGA	The Cancer Genome Atlas
TEM-1	Tumor endothelin 1
TGFβ	Transforming Growth Factor Beta
TIGIT	T Cell Immunoreceptor with Ig and ITIM Domains
TKI	Tyrosine Kinase Inhibitor
TMA	Tissue Microarray
TMB	Tumor Mutational Burden
TMTC	Transmembrane And Tetratricopeptide Repeat Containing
TP53	Tumor Protein P53
TRIO	Trio Rho Guanine Nucleotide Exchange Factor
VEGF	Vascular Endothelial Growth Factor
VGLL3	Vestigial Like Family Member 3
VMP1	Vacuole Membrane Protein 1
WNT7B	Wingless-Type MMTV Integration Site Family, Member 7B
WWTR1 (TAZ)	WW Domain Containing Transcription Regulator 1
UPA	Plasminogen Activator, Urokinase
UPS	Undifferentiated Pleomorphic Sarcoma
UPR	Unfolded Protein Response
YAP1	Yes1 Associated Transcriptional Regulator
